# Low Bone Mineral Density in Chinese Adults with Nonalcoholic Fatty Liver Disease

**DOI:** 10.1155/2013/396545

**Published:** 2013-07-31

**Authors:** Ran Cui, Hui Sheng, Xue-Fei Rui, Xiao-Yun Cheng, Chun-Jun Sheng, Ji-Ying Wang, Shen Qu

**Affiliations:** Department of Endocrinology & Metabolism, Shanghai Tenth People's Hospital, Tongji University School of Medicine, 301 Middle Yanchang Road, Shanghai 200072, China

## Abstract

*Aim*. To investigate bone metabolic characteristics in Chinese adults with nonalcoholic fatty liver disease (NAFLD). *Methods*. A total of 224 patients (99 males and 125 postmenopausal females) were recruited and divided into 4 groups: males without NAFLD, males with NAFLD, females without NAFLD, and females with NAFLD. Bone mineral density (BMD) was evaluated according to body mass index (BMI), waist circumference (WC), and serum biomarkers. **β** cell function was evaluated by HOMA2%B, HOMA2%S, and HOMA2IR. *Results*. Males in the NAFLD group had lower BMD of the right hip and the femoral neck (0.852 ± 0.117 versus 0.930 ± 0.123, *P* = 0.002; 0.736 ± 0.119 versus 0.812 ± 0.132, *P* = 0.004), and females had lower BMD of the right hip (0.725 ± 0.141 versus 0.805 ± 0.145, *P* = 0.002) even after adjusted for weight, BMI, waist, HDL, and ALT. There was no significant difference in bone metabolic markers between patients with and without NAFLD. NAFLD was an important factor that affected the bone; moreover, the effect attenuated when HOMA2IR entered into the model (*R* ^2^ = 0.160, *β* = −0.172, and *P* = 0.008). *Conclusions*. NAFLD exerts a detrimental effect on BMD in both males and females. Insulin resistance may play an important role in this pathophysiological process.

## 1. Introduction

Nonalcoholic fatty liver disease (NAFLD) is a pathological condition that encompasses a wide spectrum of liver damage, ranging from simple steatosis to nonalcoholic steatohepatitis (NASH), cirrhosis, and hepatocellular carcinoma. The pathogenesis of NAFLD is multifactorial and is characterized by overweight and insulin resistance (IR). Recently, some studies reveal that bone mineral density (BMD) is also influenced by NAFLD. Moon et al. reported a significant association between mean lumbar BMD and NAFLD in postmenopausal subjects after adjusting for age, body mass index (BMI), alanine aminotransferase (ALT), smoking status, and alcohol consumption, and they concluded that postmenopausal women with NAFLD had an increased risk of osteoporosis [1]. Pardee et al. reported that obese children with NAFLD had significantly lower BMD *Z*-scores than obese children without NAFLD; among children with NAFLD, those with nonalcoholic steatohepatitis (NASH) had a significantly lower BMD *Z*-scores than NAFLD children without NASH [2]. However, when this phenomenon is explained by overweight and insulin resistance (IR), there is a paradox. In obese subjects, it has been argued that there is a positive relationship between body weight and bone mass especially in adults [3]. However, some recent studies reveal that severe IR may negatively affect BMD [4, 5]. The role of cytokines and other bone-influencing molecules released from the inflammatory liver is still unclear. Osteocalcin (OC), which is a specific marker of bone formation, has been found to be decreased in liver with NAFLD [6, 7]. Besides its role in the calcium and bone metabolism, vitamin D may also exert pleiotropic effects in many tissues. NAFLD patients present with a marked reduction in serum 25(OH) vitamin D when compared with controls. However, although these cytokines are associated with both NAFLD and BMD, none of them have been confirmed to be involved in the process whereby NAFLD influences bone metabolism.

Considering that little is known about Chinese patients and that the mechanism underlying the relationship between NAFLD and bone defect is poorly understood, this cross-sectional study was performed in China to explore the role of overweight, IR, osteocalcin, and vitamin D in the interaction between NAFLD and BMD.

## 2. Materials and Methods

### 2.1. Subjects

Patients were recruited from the Shanghai Tenth People's Hospital of Tongji University from October 2011 to April 2012. All subjects were screened using a questionnaire on their medical history, symptoms, and findings in physical examination. Inclusion criteria were as follows: (1) subjects who were 40–70 years old and (2) males or postmenopausal females with or without NAFLD. Exclusion criteria were as follows. (1) There were other causes of chronic liver disease (hepatitis B and C, Wilson disease, *α*-1-antitrypsin deficiency, autoimmune hepatitis, and cystic fibrosis), severe alcohol abuse (>10 g of alcohol per day [8]), and drug induced fatty liver disease, especially related to amiodarone, estrogens, and vitamin A. (2) Subjects were current or former smokers who quitted smoking less than 6 months ago. (3) There were conditions known to affect bone metabolism, such as kidney, thyroid or parathyroid diseases, hyperprolactinaemia, ovary excision, rheumatoid arthritis, ankylosing spondylitis, malignant tumors, and medication with agents influencing bone metabolism (such as calcium, vitamin D, and anti-osteoporosis drugs).

A total of 99 males and 125 postmenopausal females were enrolled and divided into 4 groups according to the gender and the presence of NAFLD: males without NALFD, males with NAFLD, females without NAFLD, and females with NAFLD. This study was conducted according to the principles in the Declaration of Helsinki, and written informed consent was collected before study. The whole protocol was approved by the Ethics Committee of Shanghai Tenth People's Hospital of Tongji University.

### 2.2. Anthropometric Measurements

Weight was measured with a plethysmography scale, where patients wore minimal clothing, and height was measured with a stadiometer. Body mass index (BMI) was calculated by dividing the weight by height squared (kg/m^2^). Waist circumference (WC) was measured horizontally at the level of the umbilicus.

### 2.3. Liver Ultrasonography and NAFLD Criteria

NAFLD was diagnosed based on the ultrasonographic evidence of hepatic steatosis, such as a bright hepatic echo pattern, increased echo attenuation, and loss of intrahepatic architecture. Liver ultrasonography was conducted with a 4 MHz probe (EUB-6500, Hitachi, Tokyo, Japan) and evaluated by an experienced radiologist.

### 2.4. Dual X-Ray Absorptiometry for BMD

BMD was measured by dual-energy X-ray absorptiometry (DEXA) (Hologic (QDR-4000), USA). The BMD of lumbar spine (L), right hip (RH), and femoral neck (FN) was evaluated, and the means of L, RH, and FN served as the mean BMD (Table 4).

### 2.5. Serum Biomarkers

After overnight fasting, serum was collected. Biochemical markers, including total cholesterol (TC), triglyceride (TG), high density lipoprotein (HDL), low density lipoprotein (LDL), fasting plasma glucose (FPG), fasting insulin level (FINS), ALT, alkaline phosphatase (AKP), aspartate aminotransferase (AST), and serum creatinine (Scr) were measured with standard biochemical methods. Bone metabolic markers including type I collagen (CTX), parathyroid hormone (PTH), OC, calcitonin (CT), bone alkaline phosphatase (BAP), and 25-hydroxyl-vitamin D (25(OH)D) were measured by ELISA (Roche) according to the manufacturer's instructions. Insulin sensitivity and IR were evaluated by homeostasis model assessment 2 (HOMA2), including HOMA2%B, HOMA2%S, and HOMA2IR, calculated by HOMA2 v2.2 (http://www.dtu.ox.ac.uk/homa).

### 2.6. Statistical Analysis

Data were expressed as mean ± standard deviation (SD). Analysis of variance (ANOVA) was used to compare the baseline morphometric measurements (Table 1), BMD, and biomarkers in the control group and the NAFLD group for both genders (Tables 2 and 3). Analysis of covariance was used to adjust for weight, BMI, WC, lipid profiles, and ALT (Table 2, *P* value (a)). Multiple linear regression analysis was performed to analyze the variables associated with mean BMD and NAFLD (Table 4). Statistical Product and Service Solutions version 17.0 (SPSS 17.0) software was used for statistical analysis. A value of *P* < 0.05 was considered statistically significant.

## 3. Results

### 3.1. General Features between NAFLD Group and Control Group

At baseline, the age, height, FPG, FINS, AKP, AST, and Scr were comparable between the control group and the NAFLD group, whereas the weight, BMI, WC, lipid profiles, and ALT in subjects of the NAFLD group were markedly different from those in the control group (Table 1).

### 3.2. Comparison of BMD between the NAFLD Group and the Control Group

Subjects in the NAFLD group had significantly lower BMD in RH and FN of males and in RH of females. After adjustment for parameters (weight, BMI, WC, lipid profiles, and ALT) at baseline, a significant difference was still present (Table 2).

### 3.3. Serum Bone Metabolic Markers and HOMA Parameters

Serum bone metabolic markers including CTX, PTH, OC, CT, BAP, and 25(OH)D were comparable between the NAFLD group and the control group in both genders. Although calcium was significantly different, we did not think this difference was clinically significant. In the NAFLD group, HOMA2IR was higher in both genders, but HOMA2%S was lower in females (Table 3).

### 3.4. Correlation between Mean BMD and NAFLD

Three models were used to analyze the correlation between BMD and NAFLD taking into account all the parameters including age, BMI, waist, HOMA2IR, and NAFLD that would affect the BMD. In all the models, NAFLD played a very important role in the process of the influence on bone. Moreover, the effect attenuated when HOMA2IR entered into the model.

## 4. Discussion

In our study, subjects in the NAFLD group had significantly lower BMD than those in the control group for both genders, and the difference was still present after adjustment for variables. However, most clinical trials [1, 9] showed a negative association between NAFLD and lumbar BMD. In the present study, there was no significant difference in the lumbar BMD between the NALFD group and the control group, which might be attributed to the differences in the ethic and age. Since the BMD of femur neck and hip was not detected in previous studies, the BMD of femur neck and hip could not be compared among studies.

At baseline, subjects in the NAFLD group were characterized by higher weight, BMI, and WC, which was in accordance with other studies in which overweight or obesity was a predictor for NAFLD and other serious medical conditions [10, 11]. There is a significant relationship between BMI and relative body fat mass. Consequently, BMI is a widely accepted index of obesity, correcting for gender and ethnicity. Furthermore, there is a direct association of BMI with hepatic steatosis, NASH, and advanced liver fibrosis [12, 13]. WC is highly correlated with visceral adipose tissue (VAT) and a marker for abdominal obesity. Excess VAT, or abdominal obesity, is a risk factor of MS as compared to individuals with fat located predominantly in the lower body and subcutaneously [10]. Overweight, BMI and WC are related to bone mass to a certain extent because any increase in body mass, fat, or lean will expose the skeleton to greater forces during locomotion [14]. Bone is sensitive to, but not limited to, a variety of mechanical parameters. Holecki et al. found that the obesity failed to protect against bone mineral loss in postmenopausal women [15]. In our study, correlation analysis showed that there was no significant relation of BMD with weight, BMI, and WC. Hoy et al. speculated that the bone quality of overweight adolescents adapted to lean mass but not to greater fat mass [16], because the fat composition ratio was not significantly different between the control group and the NAFLD group.

IR was negatively associated with BMD in obese or T2DM patients [17, 18]. Even having higher bone density, as in the IR state, diabetic women have lower femoral neck strength relative to load [19]. In IR obese women, reduced IGF-1 might be an important determinant accounting for low BMD [20]. Circulating concentrations of inflammatory cytokines are reckoned to be the most important factor in causing and maintaining IR [21]. The cytokines also provide a further pathogenic link to extrahepatic organs. It has been found that IR induced by 12-week high fat diet (HFD) could impair the osteoblastic insulin signaling, osteoblast proliferation, and osteoblast survival and result in osteoporosis of the jawbone [22]. Obesity status-associated IR may induce high circulating intact (iFGF-23) and C-terminal FGF-23 (CtFGF-23), which is positively associated with PTH and negatively associated with vitamin D, resulting in low bone mass [23].

Elevated ALT and NAFLD are associated with IR, and thus, ALT has been proposed as a useful serum marker for early IR, which reflected a balance between oxidative stress and antioxidant response [24, 25]. Purnak et al. failed to find low BMD in NAFLD patients, but elevated serum ALT was found to be associated with lower BMD [26]. In our study, negative association was noted between ALT and BMD in NAFLD group, which was consistent with previously reported studies. ALT, an indicator of inflammation state in either the liver or system, will affect bone metabolism.

Osteoblasts can secrete OC, but recent work indicates that, besides acting as a specific bone marker for bone formation, OC is also associated with insulin secretion and sensitivity. After administration of recombinant OC, insulin secretion increases, blood glucose decreases, and experimental obesity is attenuated [27]. Consequently, OC has been considered to link bone to energy metabolism, although this was not evident in our study. Possible explanations for this discrepancy may be that only total serum OC was tested, whereas undercarboxylated osteocalcin (ucOC) affects energy metabolism [28]. Secondly, the association between OC and steatohepatitis is not confirmed by multivariate analysis [7]. Thirdly, decreased serum OC in Chinese NAFLD patients as compared to subjects in Western countries suggests the potential role of ethnicity [29].

Vitamin D receptors are present in several cell types and play an important role in both energy metabolism and bone metabolism [30]. 25(OH)D is a major circulating form of vitamin D and constitutes the best clinical indicator of vitamin D stores [31]. Targher et al. reported that NAFLD patients had a marked reduction in serum 25(OH)D as compared to controls, and this decrease was closely associated with the histopathological features of NAFLD [32]. However, a report on 102 NAFLD patients and 54 healthy controls showed no difference in the serum vitamin D [18], which was consistent with our results. In our study, we did not take the seasonal variation of vitamin D; since it was a cross-sectional study, we could not balance every impact factor. Moreover, vitamin D was not the only one of the factors that affect the bone metabolism; there were still other markers including type I collagen (CTX), parathyroid hormone (PTH), OC, calcitonin (CT), and bone alkaline phosphatase (BAP). Future studies are needed to clarify the correlation between vitamin D status and NAFLD.

This retrospective study still had some limitations. Firstly, being a cross-sectional study, it failed to completely exclude that some correlations were incidental. Secondly, BMD could be influenced by many factors such as physical activity, calcium/vitamin D intake, and peak bone mass, which were not evaluated in this study. Thirdly, only total OC, instead of carboxylated and uncarboxylated forms of OC, was measured. Moreover, we did not perform hepatic biopsy to evaluate the severity of NAFLD. That was because hepatic biopsy which was considered as the golden standard to diagnose NAFLD was invasive. Liver ultrasonography was also considered an effective way to investigate whether the fat existed in the liver. Further studies are needed to evaluate the effect of NAFLD on BMD in different extent. For example, we could conduct magnetic resonance spectroscopy (MRS) on the subjects or a hepatic biopsy on the animal model of NAFLD.

In conclusion, BMD is significantly decreased in elderly male and female Chinese NAFLD patients as compared to controls. Further studies are needed to identify the causative factors, especially the role of IR.

## Figures and Tables

**Table 1:** Patients' characteristics at baseline.

	Males			Females		
	Control	NAFLD	*P*	Control	NAFLD	*P*
No	53	46	—	52	73	—
Age (yrs)	58.94 ± 6.66	60.63 ± 3.68	0.130	58.75 ± 4.95	59.89 ± 6.52	0.291
Height (cm)	169.23 ± 6.40	169.70 ± 4.88	0.686	159.56 ± 5.44	157.81 ± 4.60	0.054
Weight (kg)	68.42 ± 11.94	76.32 ± 10.13	0.001*	61.16 ± 9.54	67.13 ± 15.85	0.017*
BMI (kg/m^2^)	23.81 ± 3.42	26.49 ± 3.16	<0.001*	23.98 ± 3.15	26.88 ± 5.61	0.001*
Waist (cm)	89.75 ± 10.18	96.10 ± 8.52	0.001*	88.68 ± 10.42	94.57 ± 11.14	0.003*
TC (mmol/L)	4.35 ± 1.07	4.97 ± 1.35	0.012*	5.20 ± 1.60	5.21 ± 1.19	0.985
TG (mmol/L)	1.12 ± 0.84	1.94 ± 1.64	0.002*	1.44 ± 1.19	2.27 ± 4.91	0.232
HDL (mmol/L)	1.21 ± 0.40	1.04 ± 0.26	0.012*	1.38 ± 0.40	1.25 ± 0.31	0.039*
LDL (mmol/L)	2.47 ± 0.78	2.91 ± 0.91	0.011*	3.03 ± 1.26	3.09 ± 0.92	0.728
FPG (mmol/L)	8.56 ± 3.74	8.85 ± 3.51	0.700	8.39 ± 3.78	8.67 ± 2.93	0.647
FINS (IU/L)	22.26 ± 27.63	16.74 ± 14.47	0.226	22.29 ± 41.65	23.25 ± 24.55	0.872
ALT (IU/L)	24.85 ± 24.69	30.87 ± 20.99	0.198	18.79 ± 12.29	27.49 ± 18.82	0.004*
AKP (IU/L)	73.02 ± 26.61	72.96 ± 24.61	0.990	85.06 ± 54.57	82.38 ± 30.08	0.726
AST (IU/L)	21.72 ± 11.78	23.30 ± 13.00	0.525	19.85 ± 11.69	24.12 ± 12.67	0.057
Scr (*μ*mol/L)	91.51 ± 37.10	85.239 ± 22.79	0.322	67.62 ± 29.88	66.95 ± 20.29	0.882

Data are presented as mean ± SD; NAFLD: nonalcoholic fatty liver disease; BMI: body mass index; Waist: waist circumference; FPG: fasting plasma glucose; TC: total cholesterol; TG: triglyceride; HDL: high-density lipoprotein; LDL: low-density lipoprotein; FINS: fasting insulin level; ALT: alanine aminotransferase; AKP: alkaline phosphatase; AST: aspartate amino-transferase; Scr: serum creatinine. *P < 0.05.

**Table 2:** BMD in control group and NAFLD group.

	Males				Females			
	Control	NAFLD	*P*	*P* (a)	Control	NAFLD	*P*	*P* (a)
L (g/cm^2^)	1.043 ± 0.178	0.987 ± 0.164	0.111	0.477	0.844 ± 0.150	0.812 ± 0.135	0.218	0.296
RH (g/cm^2^)	0.930 ± 0.123	0.852 ± 0.117	0.002*	0.007*	0.805 ± 0.145	0.725 ± 0.141	0.002*	0.005*
FN (g/cm^2^)	0.812 ± 0.132	0.736 ± 0.119	0.004*	0.010*	0.695 ± 0.128	0.651 ± 0.132	0.066	0.067

BMD: bone mineral density; L: lumbar BMD; RH: right hip BMD; FN: femur neck BMD; *P* value (a) in males, adjusted for weight, BMI, waist, TC, TG, HDL and LDL; *P* value (a) in females, adjusted for weight, BMI, waist, HDL, and ALT. *P < 0.05.

**Table 3:** Influence of variables on BMD in different groups.

	Males			Females		
	Control	NAFLD	*P*	Control	NAFLD	*P*
CTX (ng/mL)	0.40 ± 0.24	0.34 ± 0.23	0.158	0.44 ± 0.31	0.40 ± 0.21	0.440
PTH (pg/mL)	14.66 ± 23.45	14.17 ± 17.40	0.908	17.38 ± 23.24	16.25 ± 22.84	0.786
OC (IU/L)	12.95 ± 10.68	10.84 ± 4.53	0.568	14.61 ± 8.79	14.30 ± 5.80	0.814
CT (IU/L)	74.68 ± 51.07	87.87 ± 56.12	0.224	83.92 ± 52.69	71.47 ± 59.07	0.227
BAP (IU/L)	170.57 ± 25.45	174.89 ± 32.41	0.459	169.13 ± 23.80	170.89 ± 25.81	0.899
25(OH)D (IU/L)	47.55 ± 15.16	44.07 ± 14.45	0.248	48.52 ± 16.43	46.74 ± 24.18	0.646
Calcium (mmol/L)	2.21 ± 0.14	2.28 ± 0.12	0.017*	2.25 ± 0.13	2.30 ± 0.11	0.015*
HOMA2%B	133.96 ± 97.03	137.67 ± 82.60	0.839	115.29 ± 76.54	140.95 ± 80.59	0.076
HOMA2%S	52.50 ± 135.48	23.14 ± 13.52	0.147	76.65 ± 198.97	21.69 ± 12.49	0.020*
HOMA2IR	4.44 ± 2.41	5.41 ± 2.38	0.047*	3.94 ± 2.68	5.88 ± 2.55	<0.001*

CTX: type I collagen; PTH: parathyroid hormone; OC: osteocalcin; CT: calcitonin; BAP: bone alkaline phosphatase; 25(OH)D: 25-hydroxyl-vitamin D; HOMA2%B: homeostasis model assessment 2 of β cell function; HOMA2%S: homeostasis model assessment 2 of insulin sensitivity; HOMA2IR: homeostasis model assessment 2 of insulin resistance. *P < 0.05.

**Table 4:** Multiple linear correlation analysis between BMD and NALFD.

	*R* square	*β* coefficient	*P* value
Model 1	0.108	−0.241	<0.001
Model 2	0.120	−0.278	<0.001
Model 3	0.160	−0.172	0.008

Model 1: adjust for age, BMI, and NAFLD; Model 2: adjust for age, BMI, waist, and NAFLD; Model 3: adjust for age, BMI, waist, HOMA2IR, and NAFLD.
